# U-NTCA: nnUNet and nested transformer with channel attention for corneal cell segmentation

**DOI:** 10.3389/fnins.2024.1363288

**Published:** 2024-03-26

**Authors:** Dan Zhang, Jing Zhang, Saiqing Li, Zhixin Dong, Qinxiang Zheng, Jiong Zhang

**Affiliations:** ^1^School of Cyber Science and Engineering, Ningbo University of Technology, Ningbo, China; ^2^Institute of Biomedical Engineering, Ningbo Institute of Materials Technology and Engineering, Chinese Academy of Sciences, Ningbo, China; ^3^National Clinical Research Center for Ocular Diseases, Wenzhou Medical University, Wenzhou, China; ^4^The Eye Hospital and School of Ophthalmology and Optometry, Wenzhou Medical University, Wenzhou, China; ^5^The Ningbo Eye Hospital of Wenzhou Medical University, Ningbo, China

**Keywords:** cornea, cell segmentation, nested transformer, nnUNet, multi-scale

## Abstract

**Background:**

Automatic segmentation of corneal stromal cells can assist ophthalmologists to detect abnormal morphology in confocal microscopy images, thereby assessing the virus infection or conical mutation of corneas, and avoiding irreversible pathological damage. However, the corneal stromal cells often suffer from uneven illumination and disordered vascular occlusion, resulting in inaccurate segmentation.

**Methods:**

In response to these challenges, this study proposes a novel approach: a nnUNet and nested Transformer-based network integrated with dual high-order channel attention, named U-NTCA. Unlike nnUNet, this architecture allows for the recursive transmission of crucial contextual features and direct interaction of features across layers to improve the accuracy of cell recognition in low-quality regions. The proposed methodology involves multiple steps. Firstly, three underlying features with the same channel number are sent into an attention channel named *g*^*n*^*Conv* to facilitate higher-order interaction of local context. Secondly, we leverage different layers in U-Net to integrate Transformer nested with *g*^*n*^*Conv*, and concatenate multiple Transformers to transmit multi-scale features in a bottom-up manner. We encode the downsampling features, corresponding upsampling features, and low-level feature information transmitted from lower layers to model potential correlations between features of varying sizes and resolutions. These multi-scale features play a pivotal role in refining the position information and morphological details of the current layer through recursive transmission.

**Results:**

Experimental results on a clinical dataset including 136 images show that the proposed method achieves competitive performance with a Dice score of 82.72% and an AUC (Area Under Curve) of 90.92%, which are higher than the performance of nnUNet.

**Conclusion:**

The experimental results indicate that our model provides a cost-effective and high-precision segmentation solution for corneal stromal cells, particularly in challenging image scenarios.

## 1 Introduction

Corneal stroma layer comprises collagen fibers, accounting for 90% of the overall thickness of cornea. The corneal stroma cells, as the major cell type of the stroma, produce proteins that provide structure to the stroma and maintain the homeostasis of cornea (Barrientez et al., 2019). The injury of stromal cells tend to cause corneal irreversible damage (Barrientez et al., 2019). Previous studies have shown that the segmentation of corneal stromal cells provide the possibility to quantify cell density and other morphological changes (Arıcı et al., 2014). This process assists ophthalmologists in intuitively acquiring geometric variations to support clinical analysis (Al-Fahdawi et al., 2018). Consequently, it enables the identification of deformities or erosion caused by viruses, helping prevent irreversible pathological damage that could lead to significant visual impairment or even blindness in patients (Subramaniam et al., 2021). In particular, when compared with healthy corneas, keratoconus presents a conical protrusion and the stroma becomes significantly thinner (Lagali, 2020). Thus, the segmentation of stromal cells and the subsequent morphological measurements are helpful for ophthalmologists to judge the severity and progress of the disease.

The utility of automatic cell segmentation approaches significantly enhances the efficiency of ophthalmologists, thereby reducing the dependency on highly experienced experts (Shang et al., 2022). Various widely employed algorithms, including K-means clustering (Yan et al., 2012), edge detection (Pan et al., 2015), and watershed (Sharif et al., 2012) have been utilized to achieve automatic cell segmentation. Among them, watershed stands out due to its ability to identify challenging regions by incorporating distance transform, variance filtering, and gradient analysis (Lux and Matula, 2020). Dagher and El Tom (2008) proposed a hybrid snake-shape parameter optimization by combining the watershed algorithm with active contour, employing region merging and multi-scale techniques to alleviate issues associated with insufficient segmentation. Al-Fahdawi et al. (2018) employed Fourier transform to mitigate image noise and combined watershed for endothelial cell boundary detection. However, it is important to note that watershed approaches are prone to cause over-segmentation and often require extensive reliance on empirically tuned parameter settings.

Recent advancements in deep learning techniques provide promising possibilities for achieving more accurate cell segmentation performance. Many researchers have exploited representative networks including U-Net (Ronneberger et al., 2015), SegNet (Badrinarayanan et al., 2017), and DeepLab (Chen et al., 2017) to segment and quantify cell morphological changes. Fabijańska (2018) trained the U-Net to differentiate pixels surrounding cell boundaries and skeletons, finally obtain the segmenation results via binarizing a boundary probability map. Vigueras-Guillén et al. (2019) introduced a local sliding window in UNet and generated probability labels to enhance the contrast between positive samples and background. Subsequently, they proposed a plug-and-play attention mechanism called feedback non-local attention to assist in inferring occluded cell regions (Vigueras-Guillén et al., 2022). Given the challenges of boundary discontinuity encountered when neural networks predict ambiguous cell boundaries, some studies considered combining the advantages of CNN and watershed. Lux and Matula (2020) integrated label-controlled watershed and convolutional networks to segment densely distributed cells, incorporating segmentation function criteria to describe object boundaries.

The CNN-based models are suitable for segmenting large cells, but for cells exhibiting artifacts within their bodies, complex post-processing algorithms are essential for separating cells that are in proximity, or for reconstructing fragmented cells to form a complete cellular structure. On the other hand, the segmentation performance of CNN decreases when facing cells of different sizes within the same field of view. With the popularity of Transformer (Vaswani et al., 2017), some studies have introduced Transformer with a global perspectives to support the segmentation process (Zhang et al., 2021; Zhu et al., 2022). Zhang et al. (2021) proposed a multi-branch hybrid transformer (MBT-Net) based on edge information, which utilized Transformer and residual connection to establish long-term dependencies between space and channels. Additionally, it also incorporated body edge branches to provide edge position. Zhu et al. (2022) designed a domain adaptive Transformer for atomy aware landmark detection for multi-domain learning. Oh and Jeong (2023) introduced a diffusion model-based data synthesis method aimed at mitigating variance among nuclear classes in tasks related to cell nucleus segmentation. To alleviate the learning bias caused by artificially designed disturbances in semi-supervised models, Zhou et al. (2023) proposed a consistency training method based on wavelet to address low-frequency and high-frequency information. Wang et al. (2023) introduced a two-stage knowledge distillation method designed to prevent the accumulation of errors resulting from noise artifacts.

Previous methods frequently employed Transformer to model dependency relationships among features within the layer of same size. Simultaneously, a feature within a specific layer only interacts directly with its adjacent feature layers, making it difficult to transmit hierarchical difference information of features. This poses a challenge to integrating multi-scale information from non adjacent layers at the macro scale. Our method leverages Transformer to model the hierarchical relationships among features across different layers, with the aim of reducing the deviation and loss of edge pixels caused by interpolation and sampling between features in different layers. We recursively convey context information across different feature layers within the structure of nnUNet. This approach allows for the acquisition of high-dimensional semantic relationships between pixel points and their neighbors from various perspective. Our contributions can be summarized as follows:

- We propose a Transformer-based network called U-NTCA to segment corneal stromal cell. It integrates with dual high-order channel attention and allows for the recursive transmission of crucial contextual features to better preserve detailed cell information.
- We introduce a high-order channel attention mechanism that extends the spatial interaction among pixels from second-order to higher-order. This procedure enables feature interaction within a low computational complexity by recursively increasing the channel width.
- We design a novel transformer-based method that combines a channel attention to generate multi-scale features. This facilitates direct feature transmission across non-adjacent layers in the network.

## 2 Dataset

**Figure 1:**
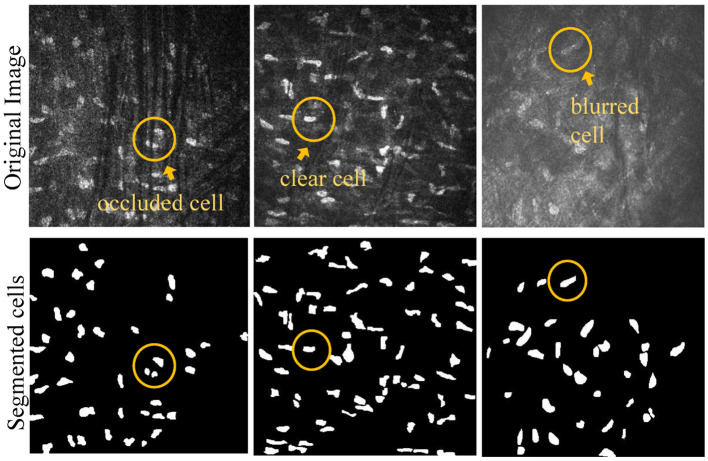
Example of different types of corneal stromal cells.

All study subjects were scanned with a laser scanning corneal confocal microscopy HRTIII (Heidelberg Engineering, Heidelberg, Germany) at the affiliated Eye Hospital of Wenzhou Medical University. The study adhered to the tenets of the Declaration of Helsinki, and was approved by the Institutional Review Board of the Affiliated Eye Hospital of Wenzhou Medical University. All the participants provided a written informed consent after receiving an explanation of the risks/benefits of the study. The dataset utilized for this study on corneal stromal cells includes 136 images, each with a resolution of 384 × 384. The training dataset contains 96 images, while the testing dataset consists of 40 images. The segmentation labels of this dataset was manually annotated by one senior ophthalmologist using the ITK-SNAP software. During training, the data augmentation operations used in the training images include rotation, increasing contrast, adding noise, translation, and flipping. This dataset comprises corneal stromal cells source from three conditions: healthy corneas (named as “normal”), corneas with keratoconus (named as “cone”), and corneas that have been eroded by viruses (named as “HSK”). In general, these cells are presented in three different types. The first type exhibits a clear field of view and clear cell structure; The second type shows that the blood vessels in the background traverse the majority of visual field, causing partial occlusion of some corneal cells. The third type of image has severe blurriness, resulting in unclear cell edge morphology. Figure 1 shows typical examples of the corneal cell dataset. Table 1 provides a detailed description of the distribution of cells of different types in the test set.

**Table 1:** The distribution of different types of cells in the test set.

	**Occlued cell**	**Blurred cell**	**Clear cell**	**All fields of view**
Normal cell	4	4	16	24
Cone	2	2	6	10
HSK	4	0	2	6
All cell types	10	6	24	40

## 3 Methodology

### 3.1 nnUNet

In medical image segmentation, researchers often develop specific algorithms tailored to address distinct research tasks and solve targeted problems. This practice, however, can result in weak generalization and robustness for general models. nnUNet is proposed to specifically solve such issues of semantic segmentation tasks in medical imaging. It places a greater emphasis on aspects such as pre-processing, training, and post-processing procedures, with a primary focus on images. By systematically modeling various configuration strategies as a set of fixed parameters (learning rate and batch size), it proves adaptable to a range of medical image segmentation tasks.

The network architecture of nnUNet is the same as that of UNet, following the encoder-decoder paradigm, which comprises a series of dense convolutional blocks. Skip connections are employed between the encoder and decoder. By concatenating the generated features for use as complementary information, efficient feature mapping occurs between internal blocks, establishing convolutional and nonlinear connections. It is noteworthy that nnUNet, aiming to enhance stability and adaptability during training while avoiding limitations imposed by batch size, substitutes the original ReLU activation functions in UNet with leaky ReLUs. In addition, it replaces the more popular batch normalization with Instance normalization. This adaptation improves nnUNet with a stronger adaptive capability, effectively resolving training instability stemming from variations in imaging methods, sizes, and voxel spacing. This enables nnUNet to be employed across a variety of scenarios.

### 3.2 U-NTCA network

**Figure 2:**
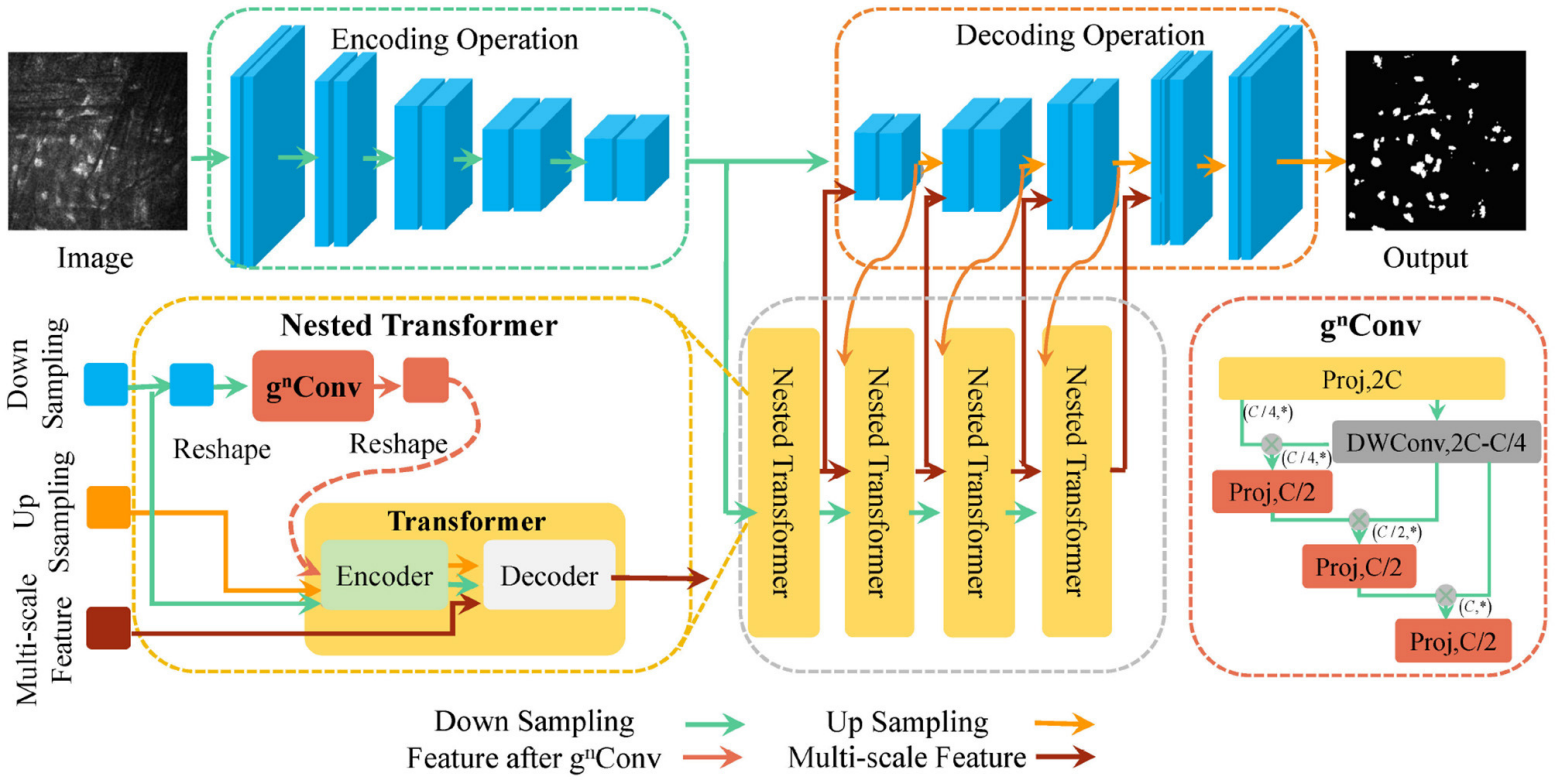
Schematic diagram of the proposed U-NTCA network.

Considering nnUNet's outstanding data processing capability and parameter adaptive adjustment, we utilize it as a backbone network and enhance it to improve information interaction between pixels and the utilization of feature information. Figure 2 shows the overall structure of the proposed U-NTCA network. First, to highlight the relationship between neighboring pixels, our focus is on the three adjacent feature layers in the UNet. For the three feature layers with the same channel, we conduct feature dimension transformations on their heights and widths. The transformed outputs are used as inputs for the proposed *g*^*n*^*Conv* channel attention, facilitating higher-order operations. This process fosters efficient interaction between neighboring pixel regions. Subsequently, the enhanced features are integrated into the current aggregated features, which are then fed into the nested transformer to aid in generating full-resolution features. Additionally, the recursive transfer of underlying feature information mitigates ambiguity and reduces information loss resulting from the sampling process.

#### 3.2.1 *g*^*n*^*Conv* high order attention mechanism

To enhance the interactive capabilities local context across varying resolutions, we introduce *g*^*n*^*Conv* module (Rao et al., 2022), which achieves explicit higher-order spatial interaction strategies within neighborhood. *g*^*n*^*Conv* is a module that implements channel attention through a combination of gated convolution and recursive strategy. It consists of three components: standard convolution, linear projections, and element-wise multiplications. It inherits the translation equivariant of standard convolution, thereby introducing inductive biases and avoiding the asymmetry arising from local attention.

Unlike the conventional approach of using *g*^*n*^*Conv* to directly interact with attention, we perform a morphological operation on feature $x_{0}\in R^{H_{0}\times W_{0}\times C_{0}}$. This involves reshaping the dimensions of width and height *x* ∈ *R*^*H*×*W*×*C*^, where $H=W=\sqrt{C_{0}}$ and *C* = *H*_0_ × *W*_0_. This strategy aims to achieve high-order interaction between global pixels across diverse fields of view. It enables the network to learn the morphological characteristics and distribution patterns from varying perspectives and directions. For transformed feature *x*, we obtain mapping feature set ϕ_*in*_(*x*) and feature auxiliary set ${\left\{q_{k}\right\}}_{k=0}^{n-1}$ with rich information embedding through the application of operation ϕ_*in*_. The operation increases the feature dimension by two times, and then divides the expanded dimension according to rule *C*_*k*_. It can be written as


$$\left[p_{0}^{HW\times C_{0}},p_{0}^{HW\times C_{0}},...,q_{n-1}^{HW\times C_{n-1}}\right]$$

$$=\phi_{in}\left(x\right)\in R^{HW\times \left(C_{0}+\sum_{0\leq k\leq n-1}C_{k}\right)}$$

Subsequently, recursively execution of gated convolution is performed, introducing the interaction between adjacent features *p*_0_ and *q*_0_ through element-wise multiplications. This process achieves a spatial mixing input function with adaptive self-attention via


$$p_{k+1}=f_{k}\left(q_{k}\right)⊙g_{k}\left(p_{k}\right),\text{k}=0,1,...,\text{n}-1$$

The channel dimension of each order can be written as


$$C_{k}=\frac{C}{2^{n-k-1}},0\leq \text{k}\leq \text{n}-1$$

Unlike the way that Transformer achieves spatial global interactions through mixing space tokens, *g*^*n*^*Conv* incrementally increases the channel width. It utilizes global computation of convolution and fully connected layers to expand the spatial interaction between pixels, progressing from second-order to higher-order interactions within less complexity.

#### 3.2.2 Transformer nested with channel attention mechanism

In nnUNet, we transmit the features processed by *g*^*n*^*Conv* module as part of multi-scale features to Transformer. For downsampling image $x_{d}\in R^{H\times W\times d}$ and upsampling image $x_{u}\in R^{H\times W\times d}$, we flatten them to generate features $x_{d}\in R^{d\times HW}$ and $x_{u}\in R^{d\times HW}$. We utilize the *g*^*n*^*Conv* to encode *x*_*u*_ and generate $g^{n}\left(x_{u}\right)$ that interacts with neighboring pixels in a high-order space. Then, *x*_*u*_, *x*_*d*_, and $g^{n}\left(x_{u}\right)$ are sent to encoder to generate enhanced ${\hat{x}}_{u}$ through self-attention.

On one hand, the upsampling feature *x*_*u*_ is sent into the encoder, accompanied by its corresponding feature $g^{n}\left(x_{u}\right)$ that has undergone spatial point multiplication to facilitate higher-order interactions. This prompts the network to devote more attention to the decisive channels, implicitly reflecting the position of cells; On the other hand, ${\hat{x}}_{u}$ could bring more semantic information by fully interacting with the multi-scale features *x*_*c*_ transmitted from lower layers in decoder, guiding *x*_*c*_ to learn the constraint relationship between pixels and their neighbors from multi-scale perspectives. This aids in the inference of missing or incorrect cell regions caused by rough interpolation process. The specific formula for the attention mechanism is given as follows


$$Attention\left(Q,K,V\right)=soft\;\max\left(\frac{QK^{T}}{\sqrt{d_{k}}}\right)V$$

The upsampling feature *x*_*u*_, downsampling feature *x*_*d*_, and enhanced feature ${\hat{x}}_{u}$ are encoded into ${\hat{x}}_{u}$. ${\hat{x}}_{u}$ contains information from higher-order pixel and their highly reliable distribution. At the same time, *x*_*u*_ also benefits from their attention interaction, creating conditions for comprehensive learning of the morphological structure and layout information of corneal cells in original image. The formula is written as follows


$${\hat{x}}_{u}=x_{u}+Attention\left(x_{u},x_{d},g^{n}\left(x_{u}\right)\right)$$

Subsequently, the joint multi-scale feature *x*_*c*_ transmitted from lower layers is updated to ${\hat{x}}_{c}$ through the cross-attention mechanism. ${\hat{x}}_{u}$ and *x*_*d*_ collaboratively guide ${\hat{x}}_{c}$ in learning the potential mapping relationship between low-resolution targets and current targets of different scales. There is a size difference between the concatenated features transmitted from the bottom layer and the current layer features. We further feed the concatenated features transmitted from the bottom layer into the decoder to interact with the current layer features, exploring the implicit correspondence between downsampling and upsampling features between adjacent layers. We pass the concatenated multi-scale features as a medium for direct interaction among different layers. This approach facilitates the discrimination capability of ambiguous pixels. The formula is given as


$${\hat{x}}_{c}=x_{c}+Attention\left(x_{c},{\hat{x}}_{u},x_{d}\right)$$

The ${\hat{x}}_{c}$ generated by *x*_*c*_ after cross attention is fed into the FFN (feedforward neural network) in residual form, which is a linear neural network with the following formula


$$FFN\left({\hat{x}}_{c}\right)=\max\left(0,{\hat{x}}_{c}W_{1}+b_{1}\right)W_{2}+b_{2}$$

The process of generating ${\tilde{x}}_{c}$ through a FFN is written as


$${\tilde{x}}_{c}={\hat{x}}_{c}+FFN\left({\hat{x}}_{c}\right)$$

We fuse the advanced multi-scale feature ${\tilde{x}}_{c}$ generated by decoder with upsampling feature of current layer in proportion to form ${\tilde{x}}_{u}$, providing more low-level local contextual information to the upsampling feature *x*_*u*_ that has information loss. ${\tilde{x}}_{u}$ is given by


$${\tilde{x}}_{u}=\alpha x_{u}+\left(1-\alpha \right){\tilde{x}}_{c}$$

#### 3.2.3 Recursive transmission of multi-scale features in U-shaped structures

**Figure 3:**
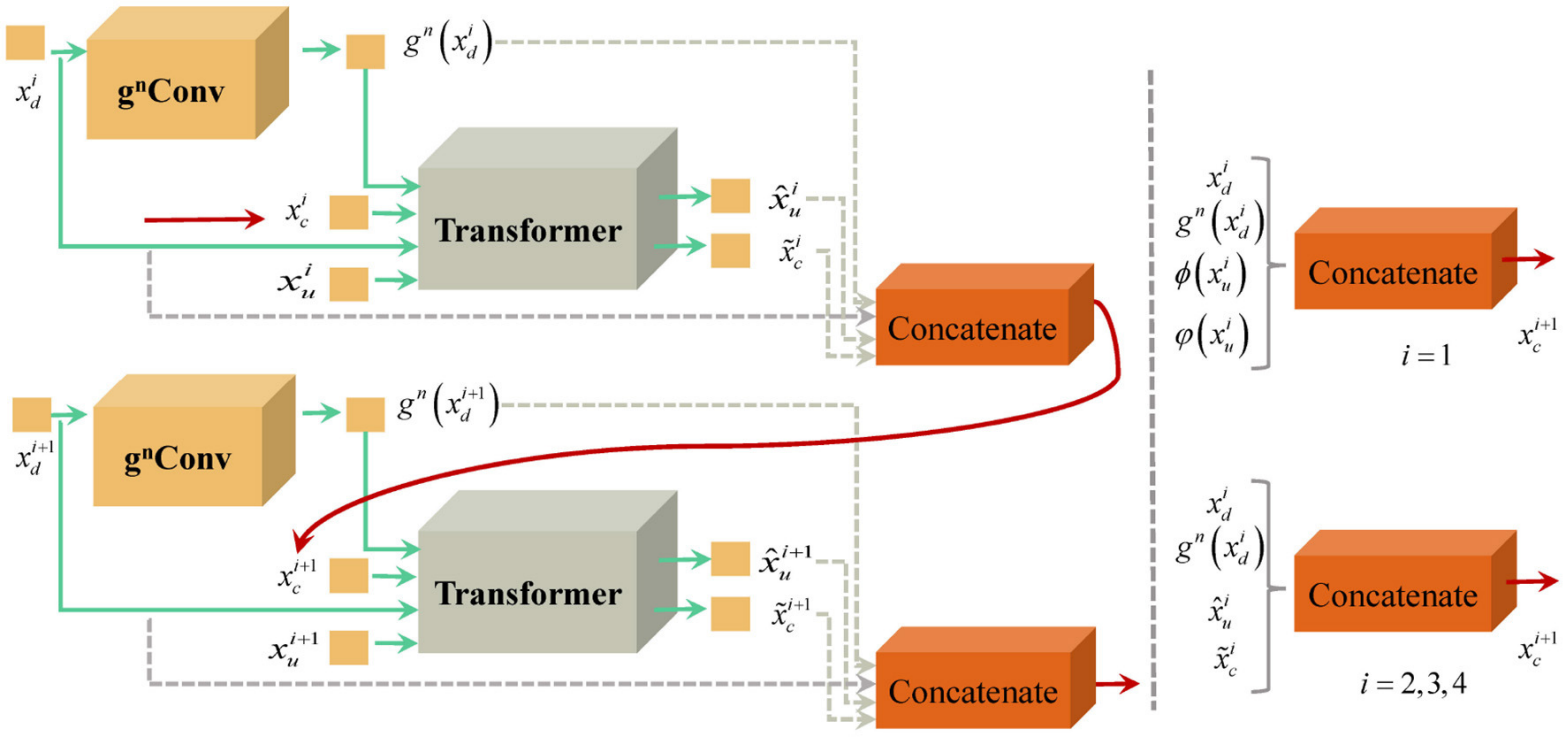
Schematic diagram of recursive transmission strategy.

We recursively implement the nested mechanism consisting of *g*^*n*^*Conv* and Transformer to deliver multi-scale features from different layers. Figure 3 displays the strategy of recursive transmission. In the process of generating upsampling features at full resolutions, we need to consider cascaded features transmitted from lower layers.

For the upsampling feature of the *i* + 1 layer, its multi-scale feature $x_{u}^{i+1}$ consists of the downsampling feature $x_{c}^{i+1}$ of the *i* layer, the advanced encoding feature $x_{u}^{i}$, the decoding multi-scale feature $x_{d}^{i}$, ${\tilde{x}}_{c}^{i}$ and $g^{n}\left(x_{u}^{i}\right)$. Thus, $x_{c}^{i+1}\left(i>1\right)$ is formulated as


$$x_{c}^{i+1}=H\left(x_{d}^{i},{\hat{x}}_{u}^{i},x_{c}^{i},g^{n}\left(x_{u}^{i}\right)\right),\text{i = 2,3,4}$$

For the lowest level features, the composition of its multi-scale features is illustrated in the following formula


$$x_{c}^{i+1}=H\left(x_{d}^{i},\phi \left(x_{u}^{i}\right),\varphi \left(x_{u}^{i}\right),g^{n}\left(x_{u}^{i}\right)\right),\text{i = 1}$$

with $\phi \left(x_{u}^{i}\right)$ and $\varphi \left(x_{u}^{i}\right)$ denote the intermediate steps in *g*^*n*^*Conv*.

Although both *g*^*n*^*Conv* and nested Transformer leverage attention to improve cell pixel segmentation, they have inherent distinctions: (1) *g*^*n*^*Conv* attention operates at a high-dimensional level, facilitating information exchange among different channels. It processes a feature internally and allocates more attention to pivotal channels to obtain a optimal combination. This method enhances the ability to distinguish pixel positions and effectively filtering out background interference; (2) To enhance the network's ability to infer positional relationships among global features and similarities between features, the Nested Transformer are inserted at the bottleneck layer of the network and functions between different aggregated features. It is connected to the decoder during the upsampling phase, progressively propagating features from different scales. This results in obtaining distribution and layout constraints of corneal cells in a 2D plane, especially for challenging cells with weak luminance and blurred boundaries.

## 4 Experiments

### 4.1 Parameter settings

The experiments were conducted using PyTorch 1.7.1 on a GeForce RTX 3090 with 24GB of RAM. For the parameterization of *g*^*n*^*Conv*, the number of iteration layers was set to *n* = 3, and the input features had a width (W) and height (H) of 22. The input feature channels followed the normal form rule 9 × 2^2*i*^(*i* = 1, 2, 3, 4). Regarding the converter network parameters, the overfitting value for the converter identification header was set to 0.1, and the forward feedback value was set to 2048. For the nested network features across different layers, the first three layers had 484 channels, and the fourth layer consisted of 256 channels. The training process employed a 5-fold cross validation method, further dividing the training and validation sets of the images in an 8:2 ratio. The fusion ratio of up-sampled features to corresponding multi-scale features was set to 3:7.

### 4.2 Evaluation metrics

In this experiment, we employ Dice, Acc, recall, pre (precision) and AUC as evaluation metrics to assess the segmentation performance. Dice quantifies the similarity between two samples, with values ranging from [0,1]. Pre (precision) denotes the proportion of correctly identified positive samples among all predicted positive samples, while recall represents the percentage of positive samples that were correctly predicted among all predicted samples. To clearly reflect the model's superior segmentation ability, Acc directly reflects the classification accuracy of the classifier. AUC quantifies the area under the ROC (Receiver Operating Characteristic) curve.

### 4.3 Comparative analysis

**Figure 4:**
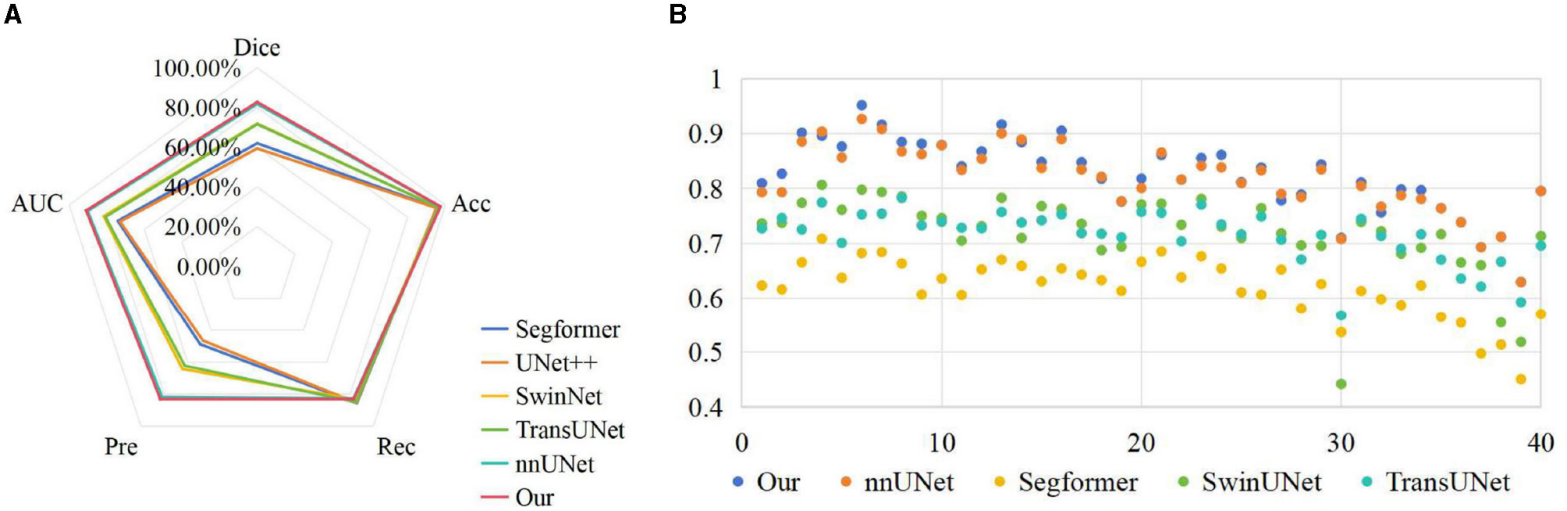
Comparison of visualization results of different methods on test set. **(A)** Comparison on various indicators; **(B)** Comparison on Dice index.

To verify the effectiveness of the proposed method, we compared the results of UNet++ (Zhou et al., 2018), Segformer (Xie et al., 2021), SwinUNet (Cao et al., 2022) and TransUNet (Chen et al., 2021) with the segmentation results of our method on the test set, as shown in Table 2. We can clearly observe that the proposed method outperforms other models in terms of all metrics. In Dice measure, the improved nnUNet reaches 82.71%, which was 23.35% higher than UNet++, 20.73% higher than Segformer, 10.85% higher than SwinUNet, 11.15% higher than TransUNet and 0.95% higher than nnUNet, respectively. Compared to nnUNet, the quantitative measurements of Dice, Acc, recall, pre, and AUC are improved by 0.08%, 0.62%, 0.55%, and 0.29%, respectively. It is demonstrated that our algorithm meets the requirement of accurate localization, thereby validating the effectiveness of the improved model. The results on the three classification datasets of Cell, HSK, and Cone intuitively show that our algorithm achieved the optimal performance on the Dice and Acc measures within these datasets. These results indicate that our method contributes comprehensively to the improvement of segmentation performance of nnUNet in multiple scenarios, rather than solving a single segmentation challenge alone. Figure 4A shows the comparison results of our method with other methods on different metrics, while Figure 4B shows the Dice values on the corneal test images of different methods. It can be intuitively seen that our method has achieved the best in all indicators, and at the same time, it outperforms other approaches in most of the test images.

**Table 2:** Comparison of experimental results of different encoding strategies for multi-scale features.

**Method**	**Normal cell**		**HSK**		**Cone**		**All**	
	**Dice (%)**	**Acc (%)**	**Dice (%)**	**Acc (%)**	**Dice (%)**	**Acc (%)**	**Dice (%)**	**Acc (%)**
UNet++	62.2	95.24	61.51	94.98	45.44	95.76	59.36	95.25
Segformer	65.02	95.49	60.34	94.97	52.55	95.97	61.98	95.43
SwinUNet	75.22	96.37	68.6	95.61	63.82	96.67	71.86	96.23
TransUNet	73.96	96.32	69.9	95.79	64.7	96.59	71.56	96.23
nnUNet	85.31	97.61	78.99	96.63	72.2	97.21	81.76	97.35
Our	**86.42**	**97.78**	**79.33**	**96.69**	**73.57**	**97.30**	**82.72**	**97.43**

Bold value indicates the best performance among all the methods in comparison.

### 4.4 Comparisons of different encoding strategies

**Figure 5:**
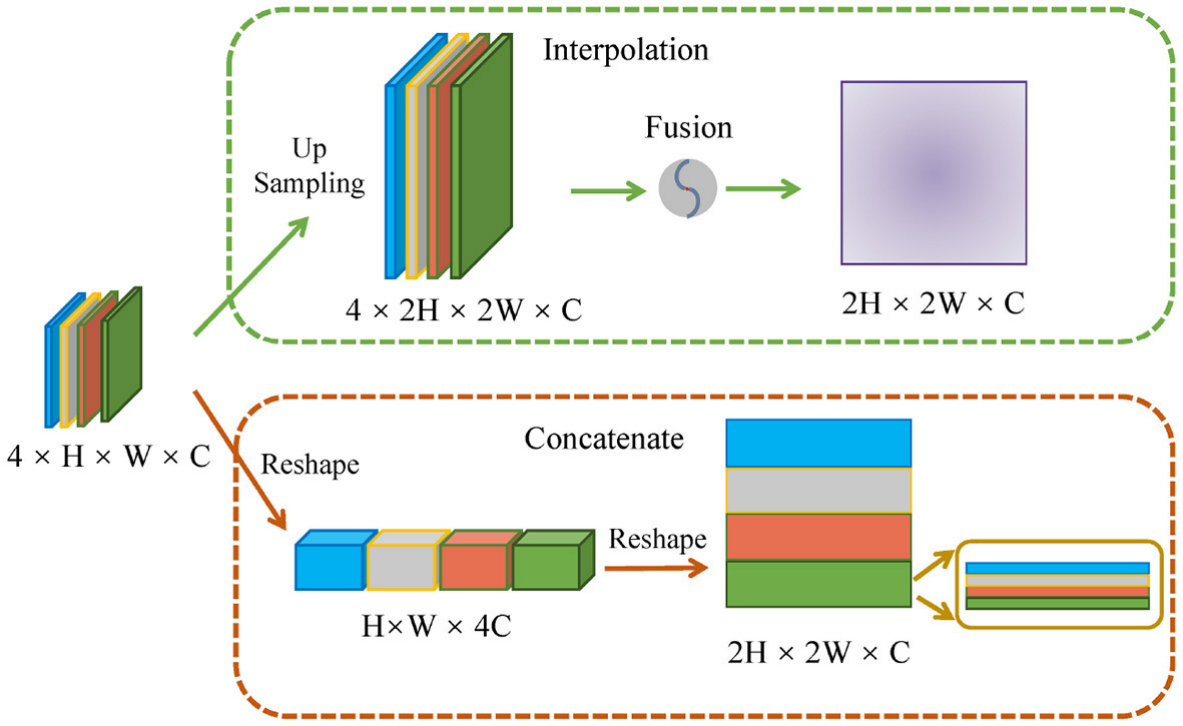
Schematic diagram of different encoding strategies.

As shown in Figure 5, we performed two comparative experiments to verify the influence of different encoding strategies of *g*^*n*^*Conv* and Transformer. To align with the dimension of high-level features, our method applied a concatenation on four smaller low-level features. In the comparative analysis in Table 3, we initially expanded the dimension of four low-level features via interpolation and then fused them with fixed proportional weights. The comparisons in Table 3 reveals that the concatenation strategy is superior to the interpolation strategy on most of the evaluation metrics. The multi-scale features based on concatenation achieve the performace of 82.72%, 97.43% and 83.06% respectively on Dice, Acc and recall. These values are respectively 0.29%, 0.11% and 2.51% higher than those achieved via interpolation. The above performance demonstrates the effectiveness of the concatenation strategy in conveying cell morphology and position distribution. This capability improves the localization of corneal cells with weaker contrast at upper layers, while the features generated through the interpolation strategy have certain information loss and ambiguous pixels, consequently diminishing the segmentation accuracy.

**Table 3:** Comparison of experimental results of different encoding strategies for multi-scale features.

	**Dice (%)**	**Acc (%)**	**recall (%)**	**Pre (%)**
Interpolation	82.43	97.32	80.54	**85.20**
Concatenation	**82.72**	**97.43**	**83.06**	83.28

Bold value indicates the best performance among all the methods in comparison.

### 4.5 Ablation experiments

The ablation experiment in Table 4 verifies the impact of *g*^*n*^*Conv* and Transformer in the proposed framework. When leveraging only *g*^*n*^*Conv* information to enhance feature interactions, Dice and AUC were increased by 0.96% and 0.46%, respectively; Moreover, by incorporating a recursive Transformer into the U-shaped architecture of nnUNet, the improved model achieved Dice and AUC values of 82.72% and 90.93%, indicating further improvements accuracy. The experimental results demonstrate that the improved nnUNet model improves the results of Dice and AUC by 0.96% and 0.76% respectively, affirming the effectiveness of the proposed method.

**Table 4:** The ablation results of different modules.

	***g* ^*n*^ *Conv***	**Transformer**	**Dice (%)**	**Acc (%)**	**Recall (%)**	**Pre (%)**	**AUC (%)**
nnUNet	×	×	81.76	97.31	82.76	81.78	90.17
our	✓	×	82.16 (+0.40)	97.36 (+0.05)	82.44 (-0.32)	82.73 (+0.95)	90.63 (+0.46)
	✓	✓	**82.72 (+0.55)**	**97.43 (+0.07)**	**83.06 (+0.62)**	**83.28 (+0.55)**	**90.92 (+0.29)**

Bold value indicates the best performance among all the methods in comparison.

### 4.6 Qualitative evaluation

**Figure 6:**
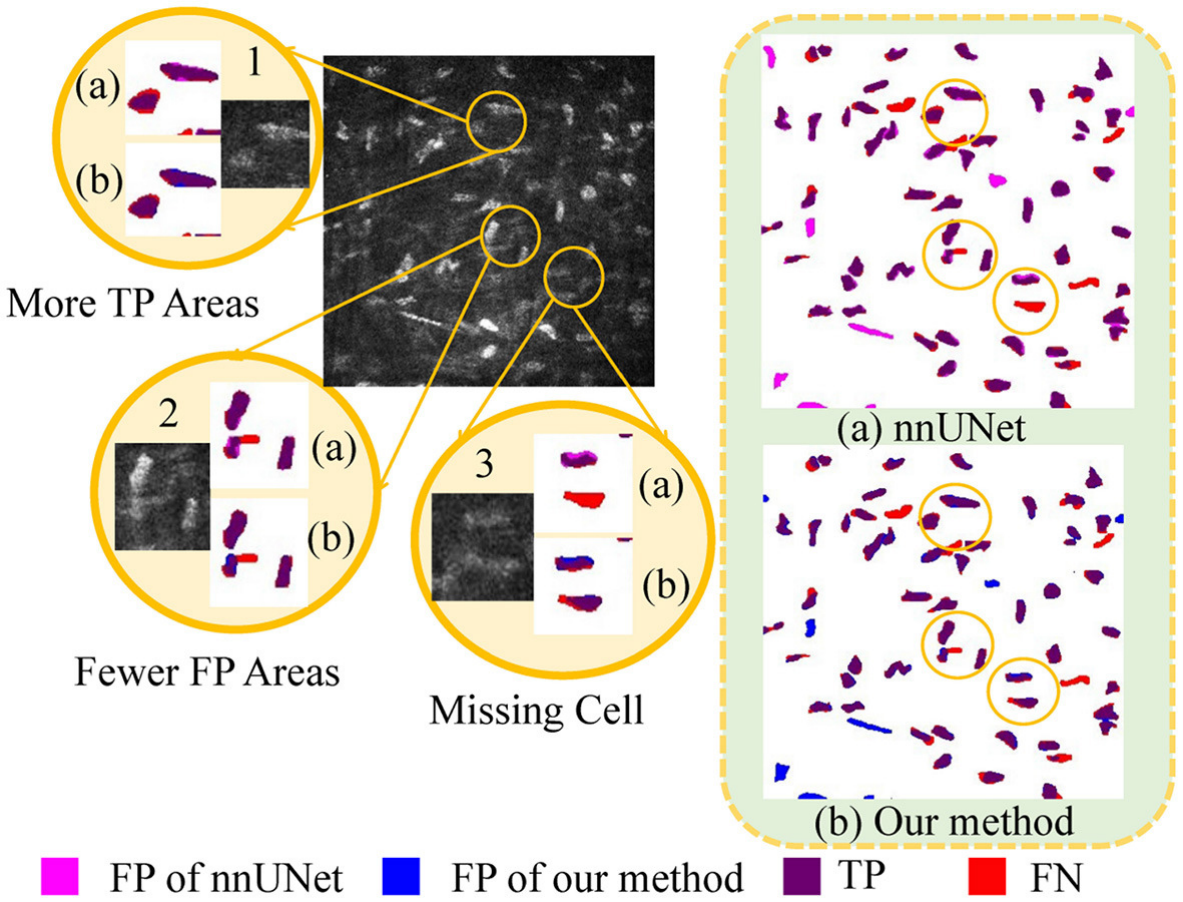
A detailed visualization comparison between nnUNet and our algorithm on local image patches.

**Figure 7:**
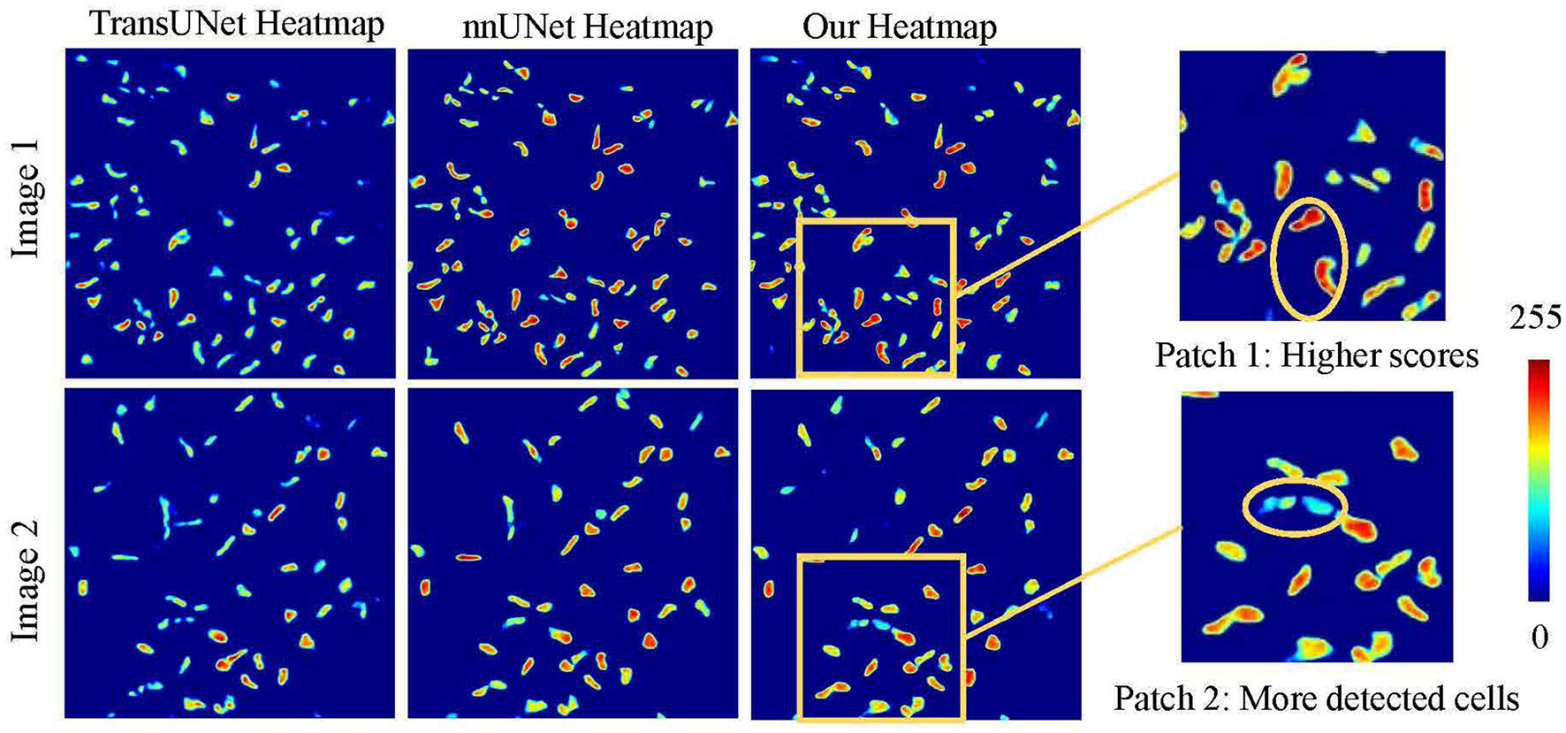
Heatmap visualization of different methods.

As illustrated in Figure 6, a detailed visualization comparison is performed between nnUNet and our method on local image patches. In Patch 1 (a), nnUNet exhibits a larger area of false positives (magenta). In Patch 2 (a), nnUNet predicted more false positive cell parts compared to our method which has a more precise detection of cell boundary in patch 2 (b). The two cells in patch 3 belong to the challenge case of low visibility. Obviously, nnUNet missed one of the corneal stromal cells, while our method which is capable of detecting both of the cells. Figure 7 visualizes the heatmap of TransUNet, nnUNet, and our method. It can be intuitively seen that the TransUNet, which is designed based on Transformer, has less cells in warm colors (such as red and yellow) compared to the other two methods. However, it shows a significantly larger number of cells in cold colors (cyan and blue). In the heatmap of nnUNet, cells are predominantly warm-colored, with clear classification boundaries for positive and negative samples. The comparison between TransUNet and nnUNet highlights the distinction between CNN and Transformer. The latter focuses on the interaction between global context, and thus it performs better at identifying more cells (in cyan) that are difficult to recognize in a blurring condition. Our algorithm effectively combines the advantages of both approaches. As demonstrated in the two zoomed patches, our method not only has high predictive scores (with more red area) for the majority of cells in patch 1, but also successfully identifies a larger number of cells (in cyan) that were overlooked by the nnUNet in patch 2.

**Figure 8:**
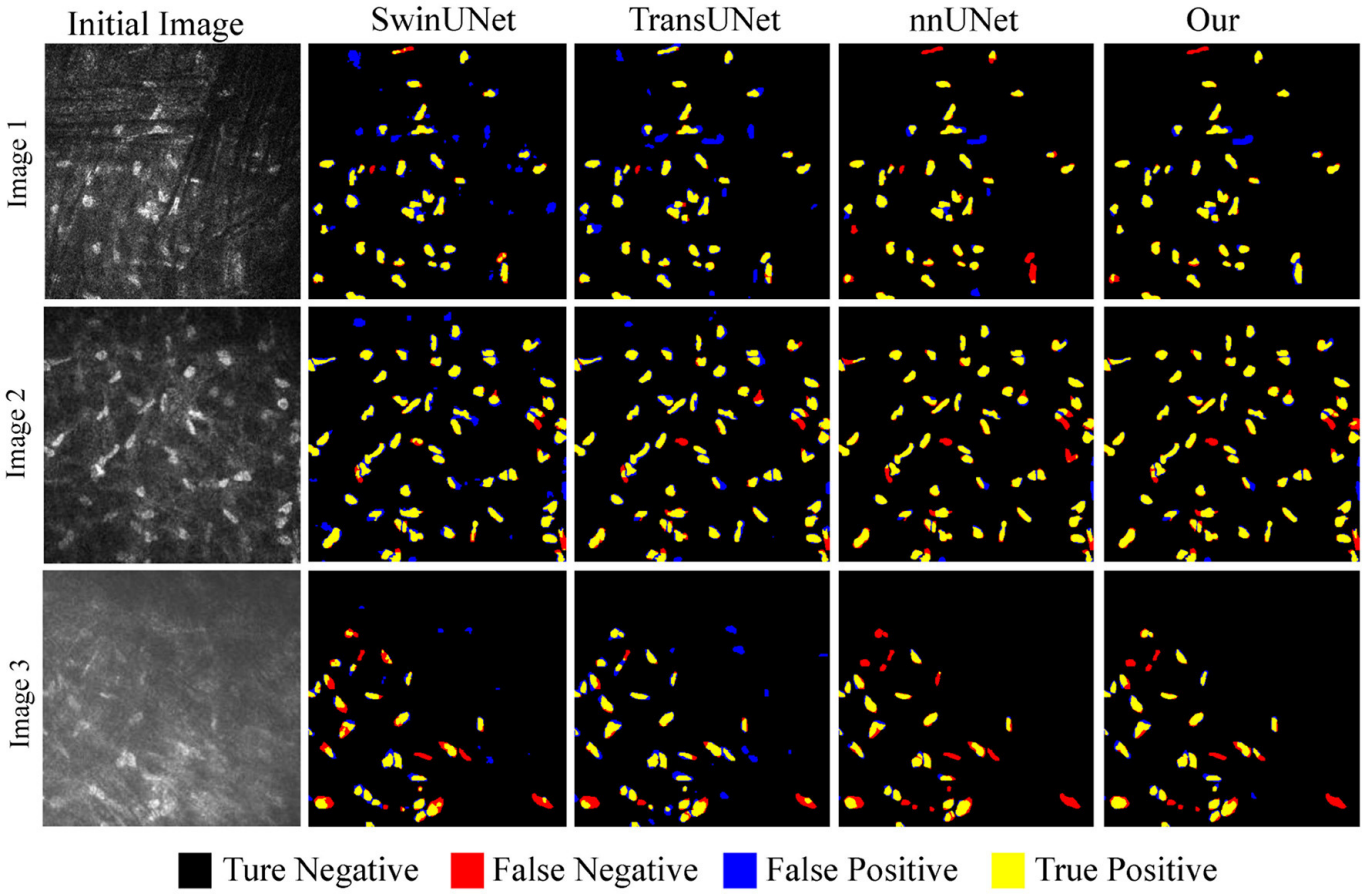
Example of overlapping results between different algorithms and real cell regions.

Figure 8 discusses the segmentation visualization results of different algorithms. In Image 1, the background vascular occlusion results in some intact cells being segmented into small fragments. nnUNet struggles to recognize some of the tiny cell fragments, whereas the proposed U-NTCA network successfully extracts the overall cell structures. Due to uneven illumination in Image 2, some cell edges are blurry with significant feature differences. This condition brings challenges for recognizing cells in dim illumination. Nevertheless, our method is able to detect more cells in low-contrast conditions. In Image 3, it can be observed that severe background interference obscures cell edges. Although the cells locate in areas with fair illumination, the accurate recognition of cell morphology and structure remains a challenging task. All the state-of-the-art approaches exhibit a notable disparity in achieving precise cell recognition, while nnUNet and our method outperforms the others in detecting a more complete cell contours.

## 5 Conclusion

The automatic and accurate segmentation of corneal stromal cells are essentially important to the rapid identification of abnormal lesions and timely prevention of the relevant diseases. To deal with the low segmentation accuracy of the existed methods under uneven illumination and occlusion, we designed a nested Transformer incorporated with nnUNet to model the implicit feature transmission across layers. The proposed model generates low-level positional and morphological features and are subsequently transmitted to upper layers to facilitate multi-scale feature fusion. In our future research, we intend to incorporate edge constraints to address challenges such as incorrectly connected cells or cells with broken edges. We will also further consider to establish a multi-task framework to achieve cell segmentation and diseases classification simultaneously, to promote computer-aided diagnosis.

## Data availability statement

The data analyzed in this study is subject to the following licenses/restrictions: Usage of the data should be under the permission of the corresponding authors. Requests to access these datasets should be directed to QZ, zhengqinxiang@aliyun.com.

## Ethics statement

The studies involving humans were approved by the Affiliated Eye Hospital of Wenzhou Medical University. The studies were conducted in accordance with the local legislation and institutional requirements. Written informed consent for participation was not required from the participants or the participants' legal guardians/next of kin in accordance with the national legislation and institutional requirements.

## Author contributions

DZ: Conceptualization, Project administration, Supervision, Writing – original draft, Writing – review & editing. JinZ: Investigation, Validation, Writing – original draft. SL: Conceptualization, Data curation, Resources, Writing – review & editing. ZD: Data curation, Resources, Writing – review & editing. QZ: Data curation, Project administration, Supervision, Writing – review & editing. JioZ: Formal analysis, Project administration, Supervision, Writing – review & editing.
